# Significance of A-waves in Isolated Calf Pain as a Manifestation of Radicular Pain During F-wave Studies: A Case Report

**DOI:** 10.7759/cureus.35254

**Published:** 2023-02-21

**Authors:** Vasily Khodulev, Aleh Kabylka, Artsiom Klimko

**Affiliations:** 1 The Functional Diagnostics Department, Republican Research and Clinical Center of Neurology and Neurosurgery, Minsk, BLR; 2 Department of Neurology, Gomel Regional Clinical Hospital, Gomel, BLR; 3 Department of Neurology, University Hospital of Zurich, Zurich, CHE

**Keywords:** nerve conduction studies, radicular pain, f-wave complex duration, f-wave duration, a-wave

## Abstract

Although isolated lower leg pain (LPP) without neurological deficit is frequently encountered in clinical practice, some of its aspects remain underexplored in the literature. There is contrasting evidence supporting the use of late responses, namely, F-waves and A-waves, in the assessment of nerve root damage. We describe the case of a 29-year-old female who presented with pain in the left calf. Neurological investigations were only significant for a positive straight leg raise test on the left side. F-wave studies of the left tibial nerve at distal and proximal points of stimulation showed the presence of the A-wave preceding the F-wave, the duration of which was prolonged. One year later, the patient reported new-onset left-sided low back pain with radiation to the gluteal area that appeared after a 10-hour airplane flight. Low back and calf pain were resolved with manipulative therapy. A-waves that had been recorded before F-waves were now no longer detectable. The presence of a neuropathic radicular component was accompanied by subclinical damage to motor fibers, as detected by routine F-waves studies. This case report illustrates the utility of integrating F-wave duration and the presence of A-waves into clinical, neurophysiological, and neuroimaging data in determining pain-generating structures in isolated LLP.

## Introduction

Back-related leg pain and isolated lower leg pain (LLP) without neurological deficit represents an important problem in clinical practice and can be caused by a slew of etiologies. Important sources of isolated LLP include various pathologic musculoskeletal processes, vascular disease, and damage to neural structures at various levels [1]. However, due to the multifaceted nature of this condition, a definitive diagnosis that relies solely on physical examination and advanced testing is not always possible. Nerve conduction studies (NCS) are uniquely positioned to allow for the assessment of the structural integrity of a peripheral nervous structure. Although NCS play a critical role in the diagnosis of a number of neurogenic disorders, there is contrasting evidence supporting the use of NCS in back-related leg pain or lumbosacral radiculopathy [2,3].

A type of late response that is often overlooked in NCS is A-waves. In this case report, we showcase the clinically relevant diagnostic utility of these NCS elements during routine F-wave studies in radicular pain manifesting as isolated neuropathic LLP. Although A-waves are present in a number of neurogenic disorders, they can also be found in healthy individuals, thus leaving their significance and generation mechanisms unclear [4-6]. This case was previously presented as a poster presentation at the 17th European Congress of Clinical Neurophysiology in Warsaw, Poland on June 8, 2019.

## Case presentation

A 29-year-old female (height 164 cm, weight 48 kg), a bank employee, presented with pain in the left calf. The patient claimed these symptoms were present since the age of 15 and were generally brief, appearing rarely (once a week) and arising when the patient leaned forward (e.g., during cleaning). There was no accompanying pain in the hips, buttocks, or the lumbar spine, and there was no history of trauma or falls. The patient repeatedly sought consultation from various medical specialists; however, no definitive diagnosis was reached. Non-steroidal anti-inflammatory drugs were prescribed, which provided short-term relief. In the last year preceding the admission to our center, the patient noted significant aggravation of her symptoms, resulting in significant morbidity. The patient described daily excruciating, persistent pain in the left calf that would sometimes be accompanied by intermittent episodes of shooting pain or electric shocks, burning, or tingling sensation that would appear in the morning after sleep, when sitting for prolonged periods, or during certain physical activity. The pain would completely subside during walking. The patient appraised the pain as ranging from 2.0 to 7.0 points and 4.0 points on the visual analog scale and the DouleurNeuropathique 4 (DN4) questionnaire, respectively.

**Figure 1:**
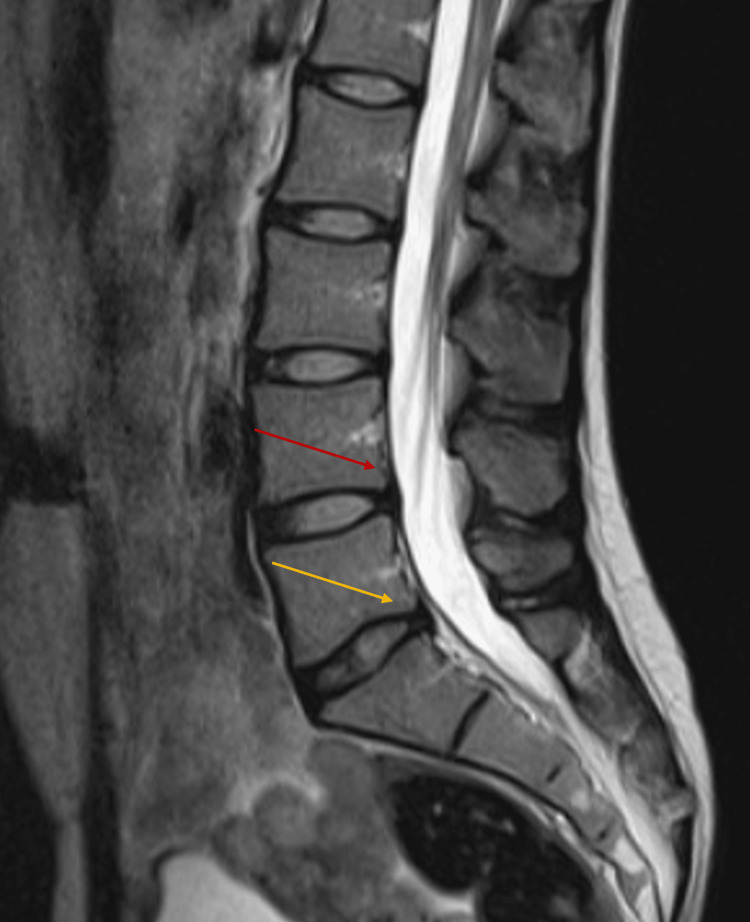
Saggital MRI of the lumbar spine showing mild dorsal protrusion of the spinal disc at L4-L5 (red arrow) and L5-S1 (orange arrow). MRI: magnetic resonance imaging

Neurological investigations were only significant for a positive straight leg raise test on the left side. Paravertebral muscles at the lumbar level had normal tone. Palpation and percussion of the spinous processes of the lumbosacral spine and paravertebral points at this level were painless. Muscle strength, sensory examination, and muscle stretch reflexes were normal. Lumbosacral magnetic resonance imaging (MRI) revealed an attenuated signal intensity at the level of the L5-S1 intervertebral disc, as well as a diffuse dorsal protrusion of the intervertebral discs L4-L5 (2.1 mm) and L5-S1 (2.3 mm). However, the sagittal diameter of the spinal canal at the level of the intervertebral discs was within normal limits (L4-L5: 14.7 mm, L5-S1: 15.0 mm) and was not suggestive of stenosis. These statements are supported by Figure 1. Other imaging investigations did not reveal any significant findings that could provide an anatomical substrate (e.g., paracentral or foraminal impingement, segment instability, or vascular claudication) that could explain the patient’s symptomatology.

**Figure 2:**
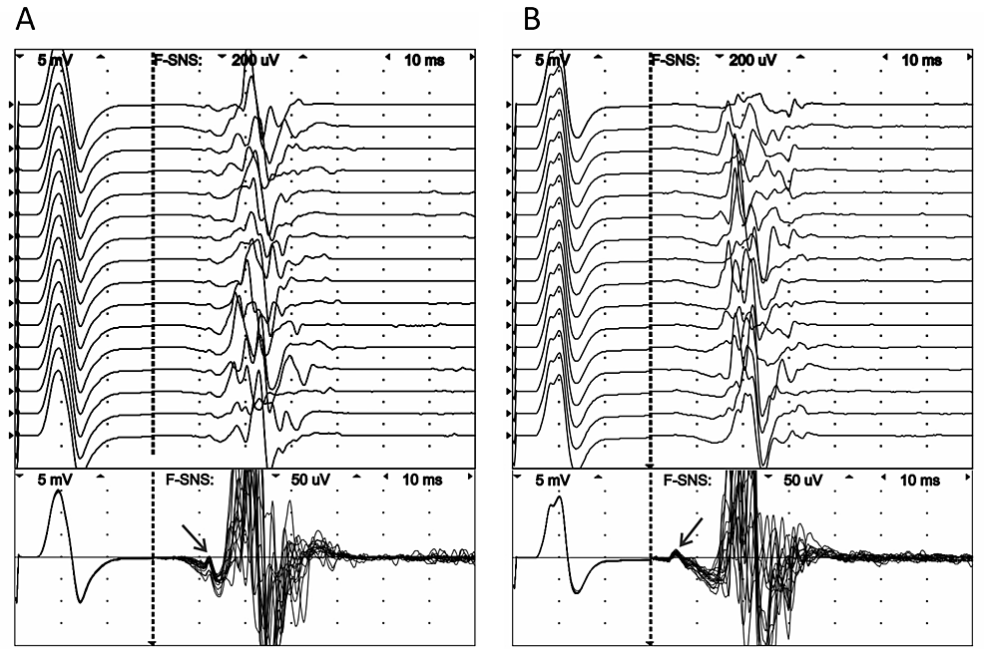
F-waves of the left (A) and right (B) tibial nerve recorded from the abductor hallucis muscle in raster (upper figure) and superimposed (lower figure) modes. The A-waves are marked by an arrow.

NCS had been performed twice within an interval of 11 days, during which the patient did not receive any treatment using a VikingSelect neurodiagnostic system (VIASYS Healthcare, USA). NCS demonstrated normal motor and sensory conduction velocities in fibular, tibial, and sural nerves. No changes were recorded from the soleus muscle in the H-reflex studies. F-wave studies at the distal stimulation point of the left tibial nerve showed the presence of the A-wave immediately before the F-wave with a latency of 42.0 ms, amplitude of 38.2 µV (from peak-to-peak), total duration of 4.0 ms, and with persistence of 100% (Figure 2A). Furthermore, an A-wave with a low amplitude (20.0 μV) and a latency of 33.6 ms was also detected on the healthy side (Figure 2B).

**Figure 3:**
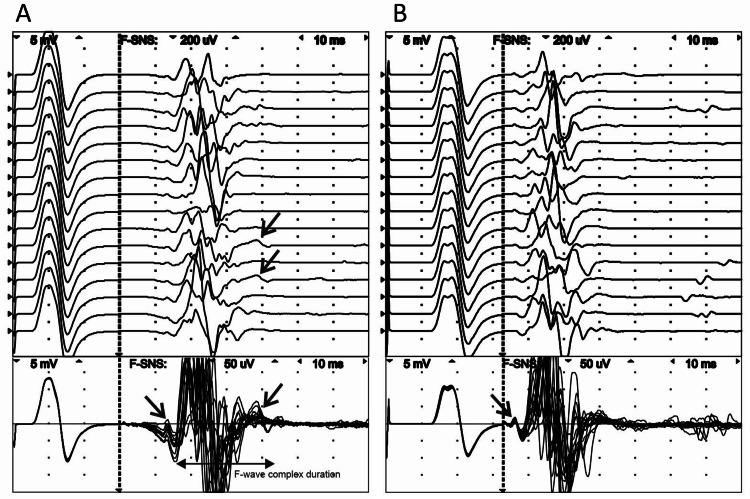
F-waves of the tibial nerve recorded from the abductor hallucis muscle at the ankle (A) and the knee (B) stimulation in raster (upper figure) and superimposed (lower figure) modes. The A-waves are marked by an arrow.

The results of the second F-wave studies of the left tibial nerve using the ankle and knee as the distal and proximal points of stimulation showed the presence of the A-wave immediately before the F-wave in proximal stimulation (Figure 3B). The shape, amplitude, and duration of the A-wave did not differ compared to the first examination. Additionally, the second A-wave was registered immediately after the F-wave with a frequency of 12.5% (2 out of 16). It had a latency of 65.4 ms, an amplitude of 65.9 μV, and a duration of 8.0 ms (Figure 3A). A-waves were not recorded during the study of the fibular nerves, where the distal and proximal stimulation points were the ankle and head of the fibula, respectively.

Among F-wave parameters, mean F-wave duration (FD) and the entire F-wave complex duration (FCD) were prolonged (Table 1). The entire FCD was defined as the time from the initial F-wave deflection (minimal F-wave latency) until the final return to baseline following the longest F-wave latency from 16 consecutive traces (Figure 3). During routine follow-up of the patient, we discovered that after a month of aquatic physiotherapy and vascular medicine treatment with diosmin for LLP, the patient reported a significant decrease in LLP, which unfortunately was not durable and recurred shortly thereafter.

**Table 1:** Results of serial F-wave studies at different times in relation to symptom onset. SD: standard deviation; ND: not done *: First and second values refer to distal and proximal stimulation, respectively. †: Value exceeded two standard deviations.

Nerve parameters	12 months		12 months and 11 days		42 months (8 months after pain disappearance)		Normal, mean ± SD
	Left	Right	Left	Right	Left	Right	
Fibular nerve							
Minimum latency, ms	44.2	44.8	47.2	ND	45.6 / 39.4*	47.0 / 39.4*	47.0 ± 3.0 / 40.1 ± 2.4
Maximum latency, ms	48.8	46.4	51.2	ND	48.8 / 41.2	48.6 / 45.8	52.2 ± 3.6 / 44.6 ± 3.1
Mean latency, ms	46.8	45.7	48.4	ND	47.1 / 40.3	47.4 / 43.0	48.9 ± 2.9 / 41.9 ± 2.5
Chronodispersion, ms	4.6	1.6	4.0	ND	3.2 / 1.8	1.6 / 6.4	5.0 ± 2.2 / 4.5 ± 0.7
Mean duration, ms	11.3	12.7	11.4	ND	11.1 / 11.7	9.9 / 11.2	9.3 ± 1.9 / 8.9 ± 1.9
Complex duration, ms	15.2	16.2	15.8	ND	16.8 / 13.8	11.8 / 19.6†	13.2 ± 1.8 / 12.5 ± 2.4
Persistence, %	81.3	43.8	56.3	ND	68.8/ 31.3	37.5/ 31.3	70.0 ± 30.7 / 77.5 ± 15.5
Tibial nerve							
Minimum latency, ms	44.6	43.4	45.4 / 38.2*	ND	45.2 / 37.6	44.6 / 36.8	47.3 ± 4.5 / 39.5 ± 2.4
Maximum latency, ms	46.8	46.2	47.4 / 39.8	ND	46.6 / 41.4	47.2 / 40.4	51.8 ± 4.2 / 43.0 ± 3.0
Mean latency, ms	45.6	44.8	46.2 / 39.1	ND	45.7 / 39.0	46.0 / 38.5	49.5 ± 4.2 / 41.2 ± 2.4
Chronodispersion, ms	2.2	2.8	2.0 / 1.6	ND	1.4 / 3.8	2.6 / 3.6	4.5 ± 1.0 / 3.5 ± 1.2
Mean duration, ms	20.2†	19.2†	20.5† / 18.3†	ND	17.8† / 17.2†	17.7† / 17.6†	11.9 ± 1.7/ 11.5 ± 1.9
Complex duration, ms	26.0†	23.8†	28.6† / 21.8†	ND	23.8† / 22.0†	21.6† / 25.2†	15.8 ± 2.1 / 14.7 ± 1.9
Persistence, %	100	100	100/100	ND	100/100	100/100	100/100

One year later, that patient reported a new onset of low back pain (LBP) with radiation to the gluteal area that appeared after a 10-hour airplane flight. The intensity of LBP varied between 3.0 and 9.0 points on the visual analog scale. There was no pain in the hip area. Non-steroidal anti-inflammatory drugs, muscle relaxants (tolperisone), shock wave therapy, massage, and acupuncture were trialed but failed to provide pain control. On the contrary, the three latter local procedures directed to the lower back exacerbated the symptoms. As a final effort after 30 months since the onset of LBP, the patient was referred to manipulative therapy, and after two sessions, LLP in the calf completely disappeared for over eight months and did not recur since. LBP rarely occurred one to two times a month and only after a long walk or strenuous physical activity. On neurological examination after eight months, the straight leg raise test remained positive on the left side only at an angle of 70 degrees, evoking a stretching pain along the posterior aspect of the thigh. Such symptoms were absent on the right side.

**Figure 4:**
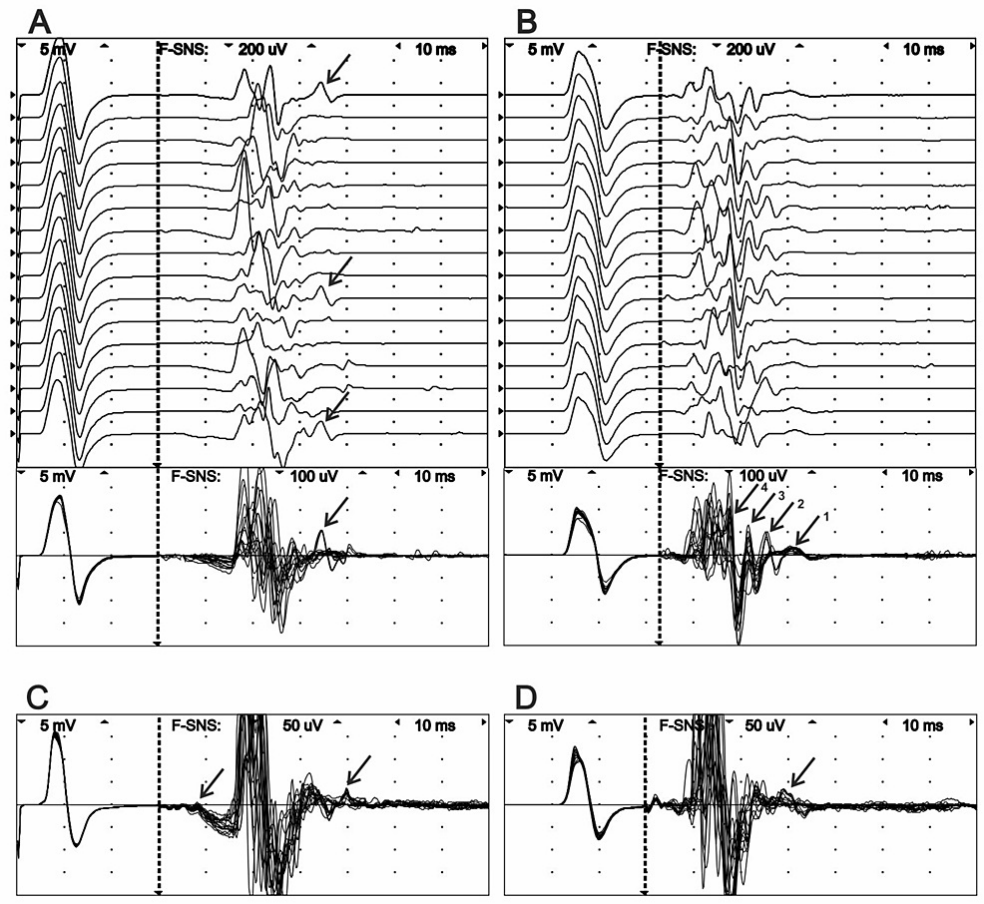
F-waves of the left (A, B) and right (C, D) tibial nerve recorded from the abductor hallucis muscle at the ankle (A, C) and the knee (B, D) stimulation in raster (upper figure) and superimposed (lower figure) modes. The A-waves are marked by an arrow.

NCS did not reveal any changes in the compound muscle action potential (CMAP); however, critically, A-waves that have been recorded previously before F-waves were now no longer detectable (Figure 4A). However, distal stimulation of the left tibial nerve revealed conserved A-waves that remained present following the F-wave with a latency of 63.9 ms (similarly to the second NCS), an amplitude of 176 μV (from peak-to-peak), duration of 5.8 ms, and persistence of 18.8 % (Figure 4A). With stimulation at the knee, A-waves were revealed following the F-waves with a latency of 58.6 ms, 53.6 ms, and possibly 49.6 and 46.6 ms (Figure 3B). We noted that A-wave #1 was not recorded in the presence of A-wave #2 and vice versa. It is probable that A-waves #3 and #4 were buried within the F-wave complex. The likelihood of their presence may be inferred from the repeatability of negative and positive peaks in superposition mode (Figure 4B, lower figure). The amplitude of these waves was 47.2 µV, 191.0 µV, 228.0 µV, and 361.0 µV, respectively. Their duration varied from 4.2 to 9.6 ms. On the right side, low-amplitude A-waves were also recorded before and after F-waves during stimulation of the distal and proximal points of the tibial nerve (Figures 4C, 4D). A-waves from the fibular nerve were not registered. Follow-up MRI did not reveal any changes or novel findings.

## Discussion

Isolated LLP without LBP is frequently encountered in clinical practice and is not sufficiently represented in the literature [7]. Differentiating between different sources of leg pain is important to make an appropriate diagnosis and identify the underlying pathology. Our patient had chronic pain with recurrent painful flares in the calf for two years with later emergence of LBP. This condition can be attributed to the *LBP and pain below the knee* or *LBP with signs of nerve root involvement* subgroups, according to the classification put forth by the Quebec spinal pain task force [8]. Classifications that would distinguish the *pain below the knee alone* subgroup within LBP or radicular pain or radiculopathy, to the best of our knowledge, do not exist. Furthermore, how its mechanistic basis relates to the natural course of disease progression, an element that plays a crucial role in the development of patient management plans, is not completely understood.

Calf pain exhibited by our patient could be characterized as neuropathic according to the DN4 questionnaire or other pain screening tools. The DN4 questionnaire has been shown to have high psychometric sensitivity and specificity to detect neuropathic pain components in patients with LBP. The relative contribution of neuropathic mechanisms was also found to increase with the degree of distal pain radiation, where pain typically manifests in the form of paroxysmal attacks [7]. In our case, the pain was provoked by taking or maintaining a certain body position and was consistent with the innervation territory of the S1 nerve root; narrowing of the left lateral recess was also present, providing a neuroanatomical basis for the pain. In addition, the positive straight leg raising test is considered important for supporting the diagnosis of radicular involvement in the form of nerve root tension or irritation but not specifically for radicular compression [1,9]. No signs of myofascial involvement or local pathologies in the lower extremity were found in our patient, just as there were no other neuropathic causes that could explain the symptoms. In our case, we noted atypical radicular pain where the atypical component was represented by neuropathic pain below the knee. The straight leg raise test was positive, the F-wave duration was prolonged, and there was the presence of A-waves during F-wave studies. Neuropathic pain is a common syndrome seen in clinical practice; furthermore, this condition is a common cause of referral for electrodiagnostic studies [10]. Patient-reported radicular pain is often vague and subjective, further complicating efficient and accurate workups given that many cases do not have evidence of nerve root compression on imaging investigations or NCS [7,11].

Although NCS are considered the gold-standard diagnostic technique for the evaluation of peripheral nerve dysfunction, it has not found notable applications in appraising lower spinal segment radiculopathy or radicular pain [2,3]. Currently, the most reported parameter is minimal F-wave latency [3]. In the current case, F-wave latency would not significantly change in response to an extended path of the motor segment of the nerve. Our patient had normal F-wave parameters, except for the FD and FCD. Research on FD is very limited when compared with the number of studies on other F-wave parameters in LBP or radiculopathies [12]. Increased FD in the context of normal latency may be caused by intact fast conduction fibers and diminished conduction in slower fibers. Furthermore, minimal latency can be maintained by preserved L5, S2, or S3 nerve roots.

The FD is determined by several factors. First, it depends on the number of motor unit potentials recruited, which is, in turn, dependent on the central excitability of motor neurons. Second, FD is influenced by the conduction property of motor fibers involved in F-wave formation. Conduction impairment can be caused by nerve fiber demyelination, conduction block, temporal dispersion, axonal degeneration, and subsequent re-innervation FD can also be influenced by the presence of A-waves, which may be recorded earlier or at the onset of F-wave formation, within the F-waves, and throughout F-wave formation [12-14]. In our case, the increase in FD occurred mainly due to the presence of A-waves at the end of the F-wave formation from both the injured and unaffected sides.

A-waves are a type of CMAP that have a constant shape and latency and can be detected during F-wave studies [4,14]. Currently, there are no recommendations on A-wave interpretation, rather only their presence or absence if described routinely [13]. They have been found in different neurogenic disorders [5,6,14-16]. A-waves are heterogeneous and can be caused by several pathophysiologic mechanisms, such as myo- or trans-axonal ephaptic transmission from one axon to another, an extra-axonal discharge elicited by an afferent action potential, ectopic discharge, and collateral sprouting during axonal regeneration [6,13,17]. A-waves have also been found to be present in up to 14-25% of lower extremity nerves in healthy individuals; however, their incidence also rises with age, leading some to speculate that A-waves may correlate with age-related degenerative changes of alpha motor neurons in the healthy population [4]. In a more recent study, Srotova et al. found the presence of A-waves to be predictive of the development of future neuropathy and radiculopathy, suggesting they represent early pathologic conduction abnormalities [6]. A-waves are more frequently observed in proximal nerve lesions but are less frequent in distal ones [5]. In the lower extremities, A-waves are commonly associated with demyelinating neuropathies and radiculopathies [5,6,13,18]. It has been suggested that demyelination is the underlying pathophysiologic correlate of the A-wave [18-20].

It is considered that in patients with proximal nerve lesions, A-wave latency is shorter than the conduction time necessary for the stimulus to travel from the site of stimulation to the site of the lesion and back. Under this assumption, the site of а nerve lesion is not the site of origin of the A-wave [13]. However, in patients suffering from cervical/lumbar radiculopathies or amyotrophic lateral sclerosis, A-waves are more frequently found to be constituents of F-waves than in any other diseases [13,14]. In our case, we registered A-waves as preceding F-waves. We noticed that A-waves were closely associated with calf pain during distal and proximal stimulation of the left tibial nerve; these A-waves were not registered after manipulative therapy (Figure 3). As such, A-waves recorded before F-waves were clinically significant and appeared to correlate with the pathologic process. However, we also registered A-waves localized after F-waves with low persistence (18.5%) on distal stimulation of the left tibial nerve after pain relief (Figure 3A). Figure 3B shows the possible presence of three or even four A-waves, a feature that was not observed during the second NCS. However, these A-waves lacked any clinical significance. Likewise, the A-waves recorded from the healthy, asymptomatic leg also lacked clinical significance (Figures 3C, 3D). Thus, A-waves may be an expression of symptomatic and latent neuropathic lesions. This observation may explain the presence of A-waves in healthy volunteers and why their incidence increases with age. The frequency of responses necessary to accept a waveform to be an A-wave has not been defined in the literature [14]. In a report by Toyokura et al., the presence of a probable A-wave before an F-wave was presented in a patient with mild S1 radiculopathy caused by intervertebral disc herniation [12].

Consequently, A-waves appear to be formed by those motor fibers whose action potentials are not susceptible to attenuation during F-wave formation and which are not blocked by action potentials emanating distally from the stimulation site or proximally from the excited motor neurons. When A-waves are part of the F-wave complex, it becomes difficult to accurately identify the onset and end of F-waves and the FD. To, at least in part, circumvent these limitations, we used the entire FCD as a novel indicator that can be rapidly evaluated in raster or superimposed modes. This indicator can account for chrono and temporal dispersion of F-waves, as well as the presence of A-waves. Lastly, the FD was also prolonged on the healthy side due to the presence of unstable A-waves.

What is the mechanism explaining the occurrence of isolated LLP and what is the relationship between pain, which is conducted along thin myelinated A-delta and unmyelinated C-fibers, and the A-wave, which reflects damage to motor fibers? A combination of factors including pressure, inflammation, and an immune response seems to be implicated in the pathogenesis of chronic radicular pain [11]. Furthermore, this combination of factors can influence sensory fibers, which represent the largest volume of spinal nerve roots. Due to the lower collagen content of nerve roots, compared to peripheral nerves, the former structures are more susceptible to deforming stress. All these factors culminate in not only nerve root inflammation and pain responses but also myelin sheath breakdown in motor and sensory fibers. At this point, the impulse can create a reflection of the stimulus or ephaptic transmission of one nerve fiber to another so that it will return to the muscle, and, hence, the neurophysiological manifestations of the motor dysfunction may then be seen in the form of an A-wave and/or an increase in FD. In addition, the convergence of different pathogenetic mechanisms can produce A-waves. In our case, an imminent factor contributing to the clinical presentation appeared to be irritation of sensory and motor fibers secondary to mechanical injury during physical exertion and certain body posture. Motor fibers were involved only at a subclinical level.

## Conclusions

Integrating analysis of the FD and the presence of A-waves into clinical, neurophysiological, and neuroimaging data can be useful in determining the pain-generating structure in isolated LLP. In our case, the presence of a neuropathic component of radicular pain was accompanied by subclinical damage to motor fibers that was detected during routine F-waves studies. Therefore, the combination of clinical data and NCS may be useful in not only diagnosing, screening, and predicting the development of LBP, radicular pain, or radiculopathy but also exploring the underlying mechanisms of neural tissue damage in such cases. This information may guide treatment selection and improve patient outcomes.

## References

[1] Vulfsons S, Bar N, Eisenberg E (2017). Back pain with leg pain. Curr Pain Headache Rep.

[2] Cho SC, Ferrante MA, Levin KH, Harmon RL, So YT (2010). Utility of electrodiagnostic testing in evaluating patients with lumbosacral radiculopathy: an evidence-based review. Muscle Nerve.

[3] Mauricio EA, Dimberg EL, Rubin DI (2014). Utility of minimum F-wave latencies compared with F-estimates and absolute reference values in S1 radiculopathies: are they still needed?. Muscle Nerve.

[4] Puksa L, Stålberg E, Falck B (2003). Occurrence of A-waves in F-wave studies of healthy nerves. Muscle Nerve.

[5] Rampello L, Rampello L, Arcidiacono A, Patti F (2020). A waves in electroneurography: differential diagnosis with other late responses. Neurol Sci.

[6] Srotova I, Vlckova E, Dusek L, Bednarik J (2017). A-waves increase the risk of developing neuropathy. Brain Behav.

[7] Vukojevic Z, Dominović-Kovačević A, Peric S, Ivo B, Sanja G, Ivana B, Dragana L (2022). Assessment of the neuropathic component in a chronic low back pain syndrome. Vojnosanitetski Pregled.

[8] Kongsted A, Kent P, Jensen TS, Albert H, Manniche C (2013). Correction: prognostic implications of the Quebec Task Force classification of back-related leg pain: an analysis of longitudinal routine clinical data. BMC Musculoskelet Disord.

[9] Vroomen PC, de Krom MC, Wilmink JT, Kester AD, Knottnerus JA (2002). Diagnostic value of history and physical examination in patients suspected of lumbosacral nerve root compression. J Neurol Neurosurg Psychiatry.

[10] Dillingham TR, Annaswamy TM, Plastaras CT (2020). Evaluation of persons with suspected lumbosacral and cervical radiculopathy: electrodiagnostic assessment and implications for treatment and outcomes (part I). Muscle Nerve.

[11] Dower A, Davies MA, Ghahreman A (2019). Pathologic basis of lumbar radicular pain. World Neurosurg.

[12] Toyokura M, Furukawa T (2002). F wave duration in mild S1 radiculopathy: comparison between the affected and unaffected sides. Clin Neurophysiol.

[13] Bischoff C, Stålberg E, Falck B, Puksa L (1996). Significance of A-waves recorded in routine motor nerve conduction studies. Electroencephalogr Clin Neurophysiol.

[14] Fang J, Cui L, Liu M (2017). A retrospective study of the characteristics and clinical significance of A-waves in amyotrophic lateral sclerosis. Front Neurol.

[15] Khodulev VI, Kabaeva KN, Stepanova JI, Shcharbina NY (2021). Severe paraproteinemic demyelinating neuropathy with impaired excitability of the distal segments of the peripheral nerves. J Clin Neuromuscul Dis.

[16] Kornhuber ME, Bischoff C, Mentrup H, Conrad B (1999). Multiple A waves in Guillain-Barré syndrome. Muscle Nerve.

[17] Magistris MR, Roth G (1992). Motor axon reflex and indirect double discharge: ephaptic transmission? A reappraisal. Electroencephalogr Clin Neurophysiol.

[18] Sartucci F, Bocci T, Borghetti D (2010). Further insight on A-wave in acute and chronic demyelinating neuropathies. Neurol Sci.

[19] Khodulev V, Ovsiankina G, Zobnina G (1998). A-wave as a sign of peripheral nervous system demyelination. Neuropathies.

[20] Rowin J, Meriggioli MN (2000). Electrodiagnostic significance of supramaximally stimulated A-waves. Muscle Nerve.

